# Voluntary Associations and Professional Reform

**Published:** 1849-01

**Authors:** Henry James Brown

**Affiliations:** Member of the American Medical Association


### VOLUNTARY ASSOCIATIONS AND PROFESSIONAL REFORM.

BY HENRY JAMES BROWN, A. M., M. D.,
*Member of the American Medical Association.*

*My Dear Sir:*

For the numbers of the Dental Register which you were obliging enough to send me, I am much indebted to you. In compliance with your request, that I should write for it, I send you a few thoughts on Voluntary Associations and Professional Reform, which I hope may at least gratify you personally, if they should not prove acceptable to your readers. They will be found, doubtless, to have a more direct pertinency to the medical than the dental profession, with the former of which, as one of its members, I may be supposed to be best acquainted; nevertheless, the intimate relation which they sustain to each other, justifies the supposition that the general principles advocated will be found to be of nearly equal importance to both.

Public sentiment demands a reformation of certain abuses of the medical and dental professions, and the unanimous voice of their membership is in harmony with this demand, but no little diversity of opinion obtains in regard to the mode of accomplishing such a work. Some are favorably impressed with the efficiency of voluntary associations as an instrumentality of reform, whilst others, willing to concede to them an influence, have less confidence in them, believing that they do not possess a power commensurate with the extent of the enterprise. The latter class conceive that they may, under favorable circumstances conduce to the mutual edification of their members, and exert a wholesome influence upon the professions, and upon general society, but feel persuaded that more is requisite than an exercise of the mere advisory powers which they possess, to remove from the professional escutcheon the foul stain which it bears. Few men, they apprehend, would regard the decisions of such bodies as authoritative. Members, indeed, might yield a courteous respect to them so long as they did not conflict with their own sense of right and justice; but what application could their obligations have to those who had never incurred them? And yet, if we are to have professional reform, it must be realized by the entire body of men regularly in the profession. Here is a difficulty, and they who claim for voluntary associations more than an advisory prerogative, have fallen into the error of attempting to make those obedient to laws who have had no part in their formation, and who have never assented to any test which may have been established. A few individuals, in a county or state, voluntarily band together as a medical association, with no other authority than that possessed by them in common with the members of a large body of professional men, and *modestly* claim to be *the* Medical Faculty, and issue their edicts with the authority of an emperor, commanding all men to choose between unconditional obedience and unmitigated disgrace! What can be more ineffably farcical than that a few members of the same family, possessing no excellency or authority above the rest, should claim to be *the* family, and exact obedience from the whole circle to such laws as they may enact, upon pain of a forfeiture of the patrimony which it was intended should descend to all! If even a majority of the medical men of our country should become thus associated, the minority would constitute a respectable body of "outsiders." Shall these bear the sobriquet of quack, or suffer other disabilities, merely because their inclination, or sense of propriety, may lead them to decline membership?

I know I shall be told that such is not the view entertained of medical and dental associations by all those who are identified with them. Very well; against the exceptions I am not inveighing, but against that class of professional men who are guilty of the folly of so thinking. An exceedingly delicate judgment is requisite to determine what *is* quackery. Who shall be the judge?—a medical or dental association? Medical quackery is not merely the violation of conventional rules, but a sin against the great principles of medical science; it is, moreover, a sin against common sense; for the principles of medicine are but a system of common sense. In their use men must think, and every man must think for himself. Medicine is essentially a manly profession, and therefore none but men can execute its functions properly; but alas, how many mere old women attempt it! Now when this very class of persons is permitted to participate in the deliberations of medical associations, being often among the moving spirits, with what justice or propriety can invidious judgment be passed upon men who desire to do their own thinking, and to operate for the good of society and the honor of the profession, in a way accordant with their own good sense, if not with the wishes of self-constituted associations. And if societies do not possess the power of conferring mind, good-breeding, and education upon those of their own members who need such gifts, how will they guard professional sanctity from the contaminating touch of ignorance, vulgarity, and dishonesty? So long as any ass or knave, who may have money to pay for it, may obtain a diploma, and be admitted into medical associations, it will be in vain for medical men to band together for self-protection. The charlatan will challenge to the competition for public favor with exulting triumph. He stands upon the broad platform of equality, upon which his diploma places him, and fights his way through all the opposition which honorable motives interpose, and, under cover of popular ignorance, achieves a victory which makes him a prince in point of fortune!

I have already hinted at the fundamental source of difficulty—the diploma. Until medical colleges are deprived of power to foist upon public confidence men who are wholly unworthy of it, it will be idle to suppose that professional reform will occur to any considerable extent. The multitude of medical institutions that have risen into being during the last ten years, have induced a vulgar competition, in its real character a species of mere jockeyism, which pays little regard to the preliminary or medical qualifications of their graduates. The correction of this evil lies directly in the way of true reform. Nothing will reach it so effectually as the enactment of a law which shall prohibit any from practicing medicine, but such as have been graduated by institutions whose standard of requirements will furnish substantial evidence that they who pass it, possess a thorough knowledge of their profession. I intend no hostility to colleges, or to voluntary associations. The former have abused their power, and the latter have, in many instances, assumed what never belonged to them. It is unjust to establish arbitrary distinctions among men who have passed the same legal ordeal of professional requirements, and it is preposterous to attempt to govern the thoughts and reason of independent practical men of education by any collusion of professional men, under the specious plea of an exclusive claim to the honors and immunities of the Medical Faculty. The united voice of an association is entitled to the respectful consideration of the whole profession, in the sense of that of any one man of respectable standing, but when it is attempted to coerce obedience, common self-respect revolts as against the pretensions of a vain man, who has quite forgotten his place. Just as a man of accomplished mind and heart may conduce to the honor and usefulness of the profession, may associations labor to effect reform. They possess an immense moral power, which, when brought to bear upon the medical profession, cannot fail to accomplish a great work. But the duty does not devolve upon them alone, nor have they exclusive power to discharge it. Upon the whole profession, regularly and lawfully in the exercise of its functions, it is incumbent to labor for its reformation. The blow must mainly be struck at the moral blight which rests upon it. An influence positively moral, if not absolutely religious, must be given to it, that the whole body medical may be aroused to a sense of honor and justice. The intrigues of party, the cabals of colleges, and the ceaseless quarrels of individual practitioners, must give place to a pervading sense of high-toned integrity. Let the profession demand, upon the part of its teachers, a character as eminent for purity as for talent, that the parent who entrusts his son to their guardianship may not feel his moral character in jeopardy, and the good work of true reform will be happily begun. This is a reasonable demand. Medical institutions are not the exclusive property of a Board of Trustees and a Faculty; they are public property also, and the public voice ought to be regarded.

Medical colleges that shall furnish thoroughly educated physicians must differ in their organization from those now in existence. I am not prepared to point out the manner and extent of that difference, but an important feature in it ought to be, that the teachers shall not reach their claims by the influence of party or clique, but by reason of superior attainments. Let the chairs be thrown open to competition, and those who show themselves best approved be the incumbents. The best men of the country are not in the medical institutions, as a general remark, but let the way be opened to them, and we shall see the Dupuytrens, the Velpeaus, and the Rouxs, come forth to take their places in the concourse. A sickly influence that pays homage to wealth, to birth, and to other factious circumstances, has too much and too constantly operated in the conferment of professional honors—the race should be more in keeping with the genius of our free institutions, having a liberal, high-minded sentiment, that looks to the greatest good of society, presiding over it. And not only should the professors hold their chairs upon the condition of superior merit, but it should be placed beyond their power to admit any aspirant to the honors of the Doctorate who could not claim them upon a similar condition. But I must not protract my remarks further. At a future time I may bestow some additional thoughts upon the subject.

In the dental profession, so far as my observation extends, the desideratum appears to be some such test as I have named, adapted of course to its peculiar wants. In avocations connected so closely with the common weal as are the medical and dental, the strong arm of the law, the guardian of social and political well-being, should promptly interfere in behalf of public health and happiness. They are professions which should not be made the medium through which designing and ignorant men may obtain a position the more effectually to render society wretched.

Truly yours,

H. J. BROWN.

PHILADELPHIA, OCTOBER, 1848.

